# The Day After: The Longitudinal Effect of COVID-19 Lockdown on Quality of Life of University Students and the Moderator Role of Mindfulness

**DOI:** 10.3390/jcm13082340

**Published:** 2024-04-18

**Authors:** Pamela Barone, Carmen Borrás-Sansaloni, Marina Ricco-Pérez, Emilio López-Navarro, Capilla Navarro-Guzmán

**Affiliations:** 1 Department of Psychology, University of the Balearic Islands, 07122 Palma, Spain; pamela.barone@uib.es (P.B.); carmen.borras@uib.es (C.B.-S.); marina.ricco@uib.es (M.R.-P.); emilio.lopez@uib.es (E.L.-N.); 2 Human Evolution and Cognition (EvoCog) Research Group, University of the Balearic Islands, 07122 Palma, Spain; 3 Behavioral Assessment and Treatment, Environmental and Laboratory Studies (BEATLES) Research Group, University of the Balearic Islands, 07122 Palma, Spain

**Keywords:** COVID-19 lockdown, quality of life, dispositional mindfulness, protective factors, university students

## Abstract

**Background:** The COVID-19 lockdown has been a major stressor for the general population, posing a considerable threat to quality of life (QoL), particularly among university students. Existing research highlights the protective role of dispositional mindfulness (DM) in mitigating stressors; however, its influence on moderating the impact of COVID-19 on QoL remains unknown. We used a longitudinal design to assess the QoL of undergraduate students before and after the COVID-19 lockdown, while also examining the potential moderating effect of DM on this impact. **Methods:** One hundred eleven Spanish undergraduate students were recruited in 2019, and 103 were followed-up in 2020. Instruments comprised a demographic questionnaire, the World Health Organization Quality of Life BREF (WHOQOL-BREF) inventory to assess QoL, and the Five Facets Mindfulness Questionnaire (FFMQ) to assess DM. **Results**: Analyses revealed statistically significant differences between the two time points in WHOQOL-BREF: Psychological, Social Relationships, and Environmental. Moderation analyses revealed that the impact of COVID-19 on WHOQOL-BREF Psychological scores was moderated by FFMQ-Observe and FFMQ-Non-judging. **Conclusions**: The COVID-19 lockdown resulted in a reduction of QoL among undergraduate students, yet this impact was moderated by DM. Specifically, present moment attention to experience (observe) and non-judgmental awareness attenuated the impact of COVID-19 on psychological well-being. Future research should focus on evaluating the protective role of preventive interventions designed to increase DM among undergraduate students.

## 1. Introduction

The World Health Organization (WHO) declared the novel coronavirus disease (COVID-19) a global pandemic on 11 March 2020 [1]. Governments worldwide, including Spain, imposed strict lockdown measures to contain the virus, which was initiated on 15 March 2020 [2]. In such circumstances, people were allowed to leave their homes only for first necessity issues. Despite these efforts, Spain faced a significant COVID-19 impact, reporting over 33,666 deaths attributed to the COVID-19 virus by 3 September 2020 [3,4,5]. Although effective in containing the spread of COVID-19, lockdown and social distancing measures highly affected people’s mental health and quality of life (QoL) [6,7,8]. Increases in psychological distress, anxiety, and depression were the most significant psychological effects reported in the literature [9,10,11,12,13]. Studies conducted in Spain have also underscored these trends [3,14], with young adults being particularly vulnerable [10,12,15,16]. A meta-analysis, encompassing 27 studies with a total of 90,879 university students across 15 countries, revealed elevated rates of anxiety (39.4%) and depression (31.2%) in this group, alongside other mental health concerns such as stress, post-traumatic stress disorder, and impaired sleep quality due to the COVID-19 pandemic [17].

QoL, as defined by the WHO [18], encompasses various aspects of well-being, including physical health, psychological state, social relationships, and environmental factors. It refers to individuals’ perception of their position in life within the cultural and value systems they inhabit, in relation to their goals, expectations, standards, and concerns. University students faced significant disruptions to their daily routines and academic pursuits, such as the transition to online classes, which may have adversely affected their QoL. Studies have shown that, during the pandemic, medical students exhibited weaker QoL in the psychological domain compared to other domains [19]. Additionally, compared to the norms of the QoL scores in the general population, psychological and social relationship QoL levels among university students were relatively low during the pandemic, while physical health and environmental QoL levels were comparable [20]. These findings underscore the significant impact of the COVID-19 pandemic on the QoL of this particular population and highlight the importance of addressing their well-being during these challenging times.

However, most of the studies, to our knowledge, are cross-sectional (due to the unforeseeable nature of the COVID-19 pandemic) or focus on a wide range of age groups. Some longitudinal studies began after the onset of the pandemic [12,21]. While these studies are crucial for comprehending the evolution of well-being and QoL among individuals throughout the pandemic, they limit the comparison within individuals before and after the onset of COVID-19. Only a handful of longitudinal cohort studies have explored changes in mental health among the same group of participants, both pre-pandemic and during the pandemic in 2020 [22].

Furthermore, it is essential to explore the potential protective factors that may have mitigated the psychological effects induced by COVID-19 previously discussed [10,23,24]. Mindfulness is defined as the individual’s inclination to direct attention and awareness to current experience [25]. Mindfulness can be considered as a state, a practice, or a trait that can be cultivated through training. On the one hand, mindfulness-based techniques have been shown efficient in reducing symptoms related to stress, anxiety, or depression [26]. During the COVID-19 lockdown, for instance, a study investigated the effectiveness of a Mindfulness-Based Stress Reduction (MBSR) program on Italian females. The results showed that MBSR training significantly improved mindfulness skills, self-acceptance, purpose in life, and relation to others. Additionally, participants with higher mindfulness scores showed greater psychological flexibility and well-being, suggesting that MBSR interventions can support psychological well-being during adverse events like the COVID-19 lockdown [6].

On the other hand, mindfulness can be regarded as a disposition, defined as an individual’s capacity and inclination to sustain mindfulness in everyday activities, embrace a nonjudgmental and accepting stance, and direct attention to present thoughts and feelings [27]. This is the focus of our study. Previous work has shown that greater subjective well-being is linked to dispositional mindfulness (DM) and is characterized by reduced levels of depression and anxiety [27]. Therefore, DM emerges as a promising candidate to help people cope with stressful situations such as the COVID-19 pandemic [28]. A study conducted on Chinese university students during the COVID-19 lockdown highlighted that enhancing DM among college students during lockdown could effectively reduce anxiety and depression while also improving subjective well-being [29]. Another study involving adults across various age groups revealed that fear of COVID-19 was associated with increased levels of depression, stress, and anxiety. Nevertheless, DM moderated this relationship [30].

While numerous studies have examined the effects that the COVID-19 pandemic has had on QoL, there remains a need for research that delves deeper into the specific mechanisms and moderators involved. Our study aims to address this gap by investigating the longitudinal impact of the COVID-19 lockdown on the QoL of university students, with a particular focus on the moderating role of DM.

Despite the growing body of literature on this topic, several significant questions remain unanswered. Firstly, existing research has primarily adopted cross-sectional designs or focused on broader age ranges, limiting our understanding of the longitudinal effects of the pandemic on QoL, especially among university students [13,17,19,20,23,24]. Secondly, while some studies have explored the relationship between mindfulness and mental health outcomes during the pandemic [28,29], few have specifically examined the protective potential of DM moderating the effect of the pandemic on QoL [30,31].

To address the gaps mentioned above, we conducted a longitudinal study with a cohort of undergraduate students before and after the onset of the pandemic. Thus, our study has two aims: first, to assess the longitudinal impact on QoL of a homogeneous sample before and after the COVID-19 lockdown, and second, to explore the role of DM as a factor that could potentially moderate the abovementioned effect on QoL.

## 2. Materials and Methods

### 2.1. Design

We designed a pre–post study following a convenience sampling of students from the first course of a public university. Data were collected during the first day of class. Data collection was conducted in September 2019, prior to the onset of the COVID-19 pandemic, and then again in September 2020, after the pandemic had begun. Data collection was conducted in person at both assessment points. Eligibility criteria were (1) proficiency in the Spanish language and (2) provision of informed consent. Data collection was gathered by the third and fourth authors. There was no economic or academic credit reward for participation in the study.

The research followed the principles of the Declaration of Helsinki and received approval from the Research Ethics Committee of the University of the Balearic Islands. Participant confidentiality was ensured by anonymizing all data collected and assigning unique identifiers to participants. Data protection measures were implemented according to institutional guidelines.

### 2.2. Sample

The sample consisted of undergraduate students enrolled in the first course of Psychology who were invited to participate in the study. At the first assessment, these students were taking an introductory subject focused on the principles and basic foundations of scientific Psychology during the first evaluation semester. Students were informed by the fourth author about the aims of the study and what their participation could entail. Once participants provided their consent to participate in the study, they started the assessment questionnaires. At the second assessment, students were taking a subject about the foundations of psychological assessment. The assessment sequence was the same as at the first assessment point and was conducted by the fourth author.

### 2.3. Instruments

Participants first completed a demographic questionnaire, providing information about their age and sex. Subsequently, the following instruments were administered:

- The World Health Organization Quality of Life: Brief Version (WHOQOL-BREF) scale to assess QoL [32]. This is a 26-item questionnaire assessing QoL across four domains: physical health (e.g., sleep quality), psychological well-being (e.g., positive emotion, self-esteem), social relationships (e.g., social support and sexual activity), and environment (e.g., transportation, healthcare assistance). Additionally, two separate items assess an individual’s overall perception of QoL and health. Responses to items are rated on a five-point Likert scale rating their QoL during the last two weeks. Domain scores are calculated by averaging the scores of items within each domain. A higher domain score indicates a better QoL in that particular domain. The WHOQOL-BREF has been widely used in the general population [33,34] and clinical samples [35,36]. The Spanish version of the WHOQOL-BREF was used, which has shown adequate psychometric properties with reliability ranging from 0.88 to 0.90 [37,38].
- The Spanish adaptation of the Five Facets of Mindfulness Questionnaire (FFMQ) to assess mindfulness in daily life [39]. The FFMQ comprises 39 items rated on a 5-point Likert scale ranging from 1 (Never or Very Rarely True) to 5 (Very Often or Always True). The FFMQ assesses trait mindfulness across five dimensions: non-reactivity to inner experience; observation of sensations, perceptions, thoughts, and feelings in the present moment; acting with awareness; describing with words emotional or affective experiences in the present moment; and non-judging of internal experiences. The FFMQ demonstrates acceptable internal consistency (0.88) as well as convergent and discriminant validity [39].

### 2.4. Statistical Analyses

Descriptive statistics were generated for both assessment points covering age, sex, and scores on the WHOQOL-BREF and FFMQ questionnaires. Before conducting any analysis, assumptions of normality and homogeneity of variances were tested through visual inspection using raincloud forests and with Shapiro–Wilk and Levene tests, as recommended by Palmer et al. [40]. 

The time assessments were compared on age, sex, WHOQOL-BREF, and FFMQ scores. Chi squared was used as a statistical contrast for categorical data, and Student t for quantitative data. We checked homoscedasticity and normality assumptions before proceeding with any manipulation check. To assess the effect of COVID-19 lockdown, we ran four ANCOVAs, one for each WHOQOL-BREF dimension, setting the years as factors and FFMQ scores as covariates. Eta squared was used as an effect size estimator. If parametric assumptions were not met, then confidence intervals were bootstrapped at 1000 samples to perform a robust statistical significance test.

If a statistically significant difference was found in the previous analyses, then a moderation analysis was performed setting FFMQ dimensions as moderators between year and WHOQOL-BREF. The aim of this analysis was to assess if DM changed the relationship between COVID-19 lockdown and WHOQOL-BREF scores. For each dimension of the FFMQ, a moderation analysis was performed, setting the remaining dimensions of the FFMQ scores as covariates. Following Hayes [41], the 16th, 50th, and 84th percentiles were calculated for each interaction below a *p*-value of 0.10. Confidence intervals in moderation analyses were bootstrapped at 5000 samples. Statistical significance was set at 5%. ANCOVA analyses and moderation analyses were conducted using SPSS 26 and the MACRO PROCESS version 4.2 [41].

## 3. Results

The sample was composed mainly of women (80.2%) with a mean age of 18.92 (*SD* = 1.44). From the original sample of 111 participants in 2019, there were 8 dropouts in 2020. These participants did not continue during the subsequent academic year. Table 1 shows the demographic features of the sample and mean scores in the FFMQ covariables.

No WHOQOL-BREF dimension met normality assumption nor homoscedasticity; Physical (Shapiro–Wilk (214) = 0.766; *p* < 0.001—Levene Test: *F*(212) = 3.94, *p* = 0.049), Psychological (Shapiro–Wilk (214) = 0.951; *p* < 0.001—Levene Test: *F*(212) = 3.18, *p* = 0.076), Social Relationships (Shapiro–Wilk (214) = 0.917; *p* < 0.001—Levene Test: *F*(212) = 7.67, *p* = 0.006), Environmental (Shapiro–Wilk (214) = 0.963; *p* < 0.001—Levene Test: *F*(212) = 9.02, *p* = 0.003). Therefore, analyses were bootstrapped to avoid bias in confidence intervals estimation. 

ANCOVA on WHOQOL-BREF Physical scores revealed no statistically significant difference for the variable year, *F*(214) = 1.42, *p* = 0.235, η^2^ = 0.007. However, significant effects were observed for the covariates FFMQ-Non-judging, *F*(214) = 6.1, *p* = 0.014, η^2^ = 0.029, and FFMQ-Acting with awareness scores, *F*(214) = 6.51, *p* = 0.011, η^2^ = 0.031, but not for FFMQ-Observe, *F*(214) = 0.14, *p* = 0.709, η^2^ = 0.001, FFMQ-Describe *F*(214) = 0.72, *p* = 0.397, η^2^ = 0.004, nor FFMQ-Non reactivity *F*(214) = 0.41, *p* = 0.523, η^2^ = 0.002.

Analysis of WHOQOL-BREF Psychological scores showed a statistically significant difference for the year factor that was associated to a nearly moderate effect size, *F*(214) = 12.3, *p* = 0.001, η^2^ = 0.057. Regarding the covariates, only FFMQ-Non-judging scores exerted a statistically significant effect on the variance accounted for the year factor, *F*(214) = 8.76, *p* = 0.003, η^2^ = 0.41. The rest of the covariates did not reach statistical significance: FFMQ-Observe, *F*(214) = 0.21, *p* = 0.647, η^2^ = 0.001; FFMQ-Describe *F*(214) = 0.91, *p* = 0.34, η^2^ = 0.004; FFMQ-Acting with awareness *F*(214) = 1.25, *p* = 0.265, η^2^ = 0.006; FFMQ-Non reactivity *F*(214) = 3.3, *p* = 0.071, η^2^ = 0.016. 

For WHOQOL-BREF Social Relationships scores, ANCOVA found statistically significant differences for the year factor, *F*(214) = 10.1, *p* = 0.002, associated to a small to moderate effect size, η^2^ = 0.047. None of the covariates exerted a statistically significant effect: FFMQ-Observe, *F*(214) = 0.88, *p* = 0.349, η^2^ = 0.004; FFMQ-Describe *F*(214) = 0.03, *p* = 0.862, η^2^ < 0.001; FFMQ-Non-judging, *F*(214) = 1.18, *p* = 0.278, η^2^ = 0.006; FFMQ-Acting with awareness *F*(214) = 0.35, *p* = 0.557, η^2^ = 0.002; FFMQ-Non reactivity *F*(214) = 0.55, *p* = 0.461, η^2^ = 0.003.

Analysis of WHOQOL-BREF Environmental scores revealed statistically significant differences for the year factor, *F*(214) = 4.81, *p* = 0.029, η^2^ = 0.023; however, no covariate was found to exert a statistically significant effect: FFMQ-Observe, *F*(214) = 0.11, *p* = 0.743, η^2^ = 0.001; FFMQ-Describe *F*(214) = 1.26, *p* = 0.262, η^2^ = 0.006; FFMQ-Non-judging, *F*(214) = 0.69, *p* = 0.408, η^2^ = 0.003; FFMQ-Acting with awareness *F*(214) = 0.27, *p* = 0.897, η^2^ < 0.001; FFMQ-Non reactivity *F*(214) = 0.02, *p* = 0.897, η^2^ < 0.001. Table 2 shows detailed information of the ANCOVA results in the main outcomes.

Moderation analyses were conducted to examine whether FFMQ scores moderate the relationship between the COVID-19 lockdown (year) and WHOQOL-BREF scores. Regarding WHOQOL-BREF Psychological scores, two models were found to be significant.

First, the model containing FFMQ-Observe significantly accounted for variance in WHOQOL-BREF Psychological scores (*R^2^* = 0.106, *F*(5, 208) = 4.95, *p* < 0.001). Furthermore, the interaction between the factor and the moderator was statistically significant (*t* = 2.43, *p* = 0.016; see Table 3). Interaction analysis revealed that at low levels of FFMQ-Observe (16th and 50th percentiles), a statistically significant negative effect of the COVID-19 lockdown on WHOQOL-BREF Psychological scores was observed (*β* = −1.71, *t* = −4.07, *p* < 0.001 and *β* = −0.9, *t* = −3.18, *p* = 0.002, respectively). Conversely, at high levels of FFMQ-Observe scores (84th percentile), no statistically significant effect of the COVID-19 lockdown on WHOQOL-BREF Psychological scores was observed (*β* = −0.32, *t* = −0.83, *p* = 0.406). 

Second, the model containing FFMQ-Non-judging significantly accounted for variance in WHOQOL-BREF Psychological scores (*R*^2^ = 0.169, *F*(5, 208) = 8.43, *p* < 0.001). Furthermore, the interaction between the factor and the moderator was statistically significant (*t* = 3.45, *p* = 0.001; see Table 3). Interaction analysis revealed that at low levels of FFMQ-Non-judging (16th and 50th percentiles), a statistically significant negative effect of the COVID-19 lockdown on WHOQOL-BREF Psychological scores was observed (*β* = −1.95, *t* = −4.97, *p* < 0.001 and *β* = −1.95, *t* = −4.97, *p* < 0.001, respectively). In contrast, scores above the 84th percentile did not moderate the effect of the COVID-19 lockdown on WHOQOL-BREF Psychological scores (*β* = −0.1, *t* = −0.28, *p* = 0.779).

**Figure 1:**
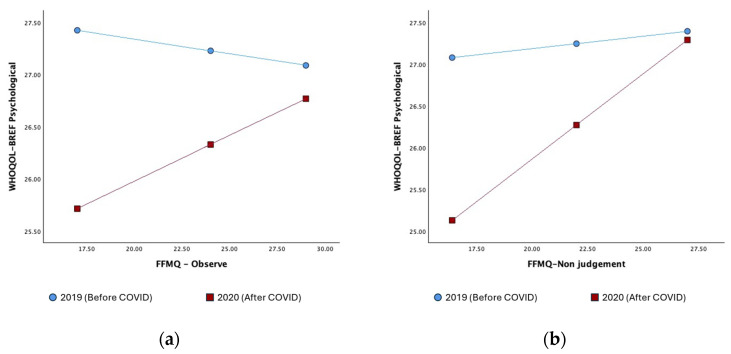
Moderation effect of the FFMQ-Observe (**a**) and of the FFMQ-Non-judging (**b**) scores on the impact of year on WHOQOL-BREF Psychological scores.

Table 3 displays the results of the moderation analysis examining the interaction between the dimensions of the WHOQOL-BREF and FFMQ scores. Figure 1 shows the moderation effects of the FFMQ scales (Observe and Non-judging) on the impact of year on WHOQOL-BREF Psychological scores.

## 4. Discussion

While numerous studies have examined the immediate effects of the pandemic on various populations [7,8,10,11,15,42,43], there has been a gap in research regarding how QoL changed within a specific cohort before and after the onset of the pandemic. In our longitudinal study, we assessed the QoL of university students before and after the COVID-19 lockdown, while also investigating the potential moderating effect of DM on this impact. Our findings revealed two main points. First, we observed a decline in the QoL of university students following the lockdown, particularly in the psychological, social relationships, and environmental dimensions of QoL. Second, we found that DM played a moderating role in the relationship between the COVID-19 lockdown and psychological QoL. Specifically, the Observe and Non-judging dimensions of the FFMQ were identified as moderators. This study contributes to a more nuanced understanding of how the pandemic influenced different aspects of QoL among university students.

This study makes several important contributions to the existing literature on the effects of COVID-19 lockdown on university students’ QoL. Our findings align with previous research indicating that the COVID-19 pandemic has had a significant negative impact on various dimensions of QoL among university students [19,20]. For instance, Chawla [19] indicated that medical students also experienced lower QoL during the pandemic, particularly in the psychological dimension. Furthermore, declines in both psychological and social relationship QoL levels among university students during the pandemic have been reported [20]. Additionally, our study also contributes to this discussion by showing the impact of the pandemic on the environmental dimension of QoL. In conclusion, the significant impact of the COVID-19 pandemic on QoL is undeniable. To account for these findings, the widespread prevalence of depression, anxiety, and stress documented during the pandemic [9,10,11,12,13,17,19,20] likely played a significant role in the decline of the psychological dimension of QoL among participants in our study. Similarly, the implementation of social distancing measures and restrictions on social activities to mitigate the spread of COVID-19 may have provoked the decrease in social relationship QoL. Additionally, the environmental dimension of QoL, which encompasses various factors, like financial resources, safety, and access to health services [44], may have been adversely affected by the pandemic-related restrictions and changes, leading to a decline in this domain as well.

Our findings point to a protective effect of DM on the effect of COVID-19 lockdown on QoL. In particular, individuals with high levels of observation and nonjudgmental awareness did not experience a statistically significant impact of the COVID-19 lockdown on psychological well-being (i.e., WHOQOL-BREF Psychological scores). We found that people with a strong tendency to notice or attend to stimuli in one’s internal and external environment resulted in a lesser negative effect of COVID-19 lockdown than individuals who did not show that tendency. Also, our results suggest that people that tend to accept internal experiences, such as thoughts, feelings, and sensations, without judging them showed a lesser negative effect on psychological well-being. These results are convergent with studies reporting the protective effect of these tendencies in a wide range of stressful situations that reduce psychological well-being, such as breast cancer [45], psychotic experiences [46], stressful events in adolescence [47], suicidal ideation [48], adverse childhood experiences [49], or trauma [50]. Furthermore, our results validate and deepen previous studies suggesting that DM moderates the adverse psychological effects of COVID-19. Previous research has linked an increase in subjective well-being with DM, resulting in a reduction in symptoms of depression and anxiety [27,51]. This capacity of mindfulness to confront stressful situations has become particularly evident during the COVID-19 health crisis [28]. Enhancing DM in Chinese university students effectively mitigated anxiety and depression while simultaneously improving subjective well-being [29]. Consistently, our findings highlight the protective potential of DM in buffering the adverse impact of the pandemic on QoL. This underscores the relevance of cultivating mindfulness skills, particularly during periods of heightened stress and uncertainty, such as during COVID-19 confinement. 

The growing evidence regarding the protective role of DM in psychological COVID-19 impact can be explained by the Monitor and Acceptance Theory [52]. The Monitor and Acceptance Theory posits that the positive effect exerted by dispositional mindfulness on psychological QoL accounts for the combination of two components: attention monitoring and acceptance. The attention monitoring component involves the self-regulation of attention to notice distractions and disengage from them to refocus on the present. The acceptance component alludes to an open, nonjudgmental stance towards whatever arises in the present moment. It is not merely toleration but an active, welcoming approach to experiencing thoughts, feelings, and sensations without trying to change them [53]. Consequently, attention monitoring focused on the present is associated with psychological well-being only when one’s experiences are approached with acceptance. According to Simione and Saldarini [54], the Monitoring component can be assessed with FFMQ-Observe and the Acceptance component with FFMQ-Non-judging. We found that both dimensions moderate the negative effect on psychological QoL of the COVID-19 lockdown. In the context of our study, we can surmise that an individual who was aware of the implications of the COVID-19 lockdown yet refrains from evaluative judgment would exhibit a better psychological QoL than another individual who, despite being aware of the implications, dwells on the potential consequence.

Overall, our findings have significant implications for university students and relevant stakeholders, especially in facing similar challenging circumstances in the future [13]. Given the observed decline in QoL dimensions, prioritizing mental health support for university students is essential. This may involve establishing psychological support programs that offering counseling services, workshops on stress management, and resilience-building activities [23]. Ensuring easy access to mental health resources and encouraging students to seek help when needed is also crucial. Bearing in mind that the effect of COVID-19 on QoL occurred in different stages of life—like childhood and adolescence [55], elderly people [56], and pregnant women [43]. Also, in a university setting the implementation of interventions fostering dispositional mindfulness could have side benefits given the mediator effect of DM on the relationship between anxiety and exam performance [57].

While our study provides valuable insights, several limitations should be taken into consideration. First, our sample comprised psychology students, which may limit the generalizability of the findings to other university students or broader populations. Second, the uneven distribution of sex in our sample, due to the overrepresentation of women in psychology programs, could potentially impact the generalizability of the findings. Specifically, research by Trzcionka et al. revealed differing responses to stressful situations among male and female students, with women in technical fields exhibiting lower resistance and those in medical sciences displaying higher levels of resilience [58]. Third, the sample size was relatively small, potentially limiting the statistical power of the analysis. Future studies should be aimed at exploring gender differences and include more diverse and larger samples to enhance the generalizability of these findings and provide insights into the nuanced effects of mindfulness on QoL across different populations. 

The limitations mentioned above highlight the need for caution when interpreting the findings and, at the same time, suggest avenues for future research to address these concerns. Further studies should aim to explore the potential role of mindfulness in enhancing psychological QoL during global events involving drastic changes or significant consequences. Given the unpredictable nature of pandemics, replicating our findings using a longitudinal design may not be feasible. Researchers could evaluate the effectiveness of preventive interventions designed to enhance DM among university students and assess their protective role in maintaining psychological well-being during challenging times.

## 5. Conclusions

Our study emphasizes the enduring impact of the COVID-19 pandemic on the QoL among university students, as evidenced by declines in the psychological, social relationships, and environmental dimensions. Additionally, it highlights the potential moderating influence of DM in alleviating the negative impact of the pandemic and fostering psychological well-being. 

## Figures and Tables

**Table 1:** Demographic features of the sample.

Outcome	2019 (*n* = 111)	2020 (*n* = 103)	Statistics
Age (mean, *SD* ^1^)	18.92 (1.44)	19.9 (1.41)	*F* = 25.66 *p* < 0.001
Sex (*n*, %)			
Man	22 (19.8)	19 (18.4)	*χ^2^* = 0.06 *p* = 0.799
Woman	89 (80.2)	84 (81.6)	
FFMQ (mean, *SD*)			
Observe	23.48 (5.86)	23.58 (6.02)	*F* = 0.02 *p* = 0.897
Describe	24.41 (2.97)	24.43 (3.14)	*F* = 0.01 *p* = 0.976
Non-judging	21.81 (6.11)	22.13 (4.93)	*F* = 0.17 *p* = 0.68
Acting with awareness	23.89 (5.48)	23.95 (6.02)	*F* = 0.01 *p* = 0.94
Non reactivity	19.68 (4.03)	19.65 (4.1)	*F* = 0.01 *p* = 0.951

^1^ *SD*: standard deviation.

**Table 2:** ANCOVA results for the effect of year in WHOQOL-BREF scores while controlling for FFMQ scores.

WHOQOL-BREF	2019	2020	Statistics		
	*Mean (SD)*	*Mean (SD)*	*F*	*p*	*Eta*
Physical	33.29 (2.14)	33.02 (1.35)	1.42	0.235	0.007
Psychological	27.24 (1.92)	26.29 (2.28)	12.3	0.001	0.057
Social Relationships	13.09 (1.47)	12.38 (1.79)	10.1	0.002	0.047
Environmental	34.68 (2.97)	33.64 (3.86)	4.81	0.026	0.023

**Table 3:** Moderation analyses of the interaction between outcome (WHOQOL-BREF) and moderator (FFMQ dimensions) variables.

WHOQOL-BREF	FFMQ Dimensions	*β*	IC 95%	*t*	*p*
Psychological	Observe	0.116	0.022, 0.21	2.43	0.016
	Describe	0.06	−0.124, 0.245	0.645	0.52
	Non-judging	0.174	0.075, 0.274	3.448	0.001
	Acting with awareness	0.069	−0.029, 0.167	1.385	0.168
	Non reactivity	0.012	−0.127, 0.151	0.176	0.861
Social	Observe	0.023	−0.052, 0.098	0.606	0.545
Relationships	Describe	−0.031	−0.176, 0.115	−0.417	0.677
	Non-judging	0.058	−0.024, 0.14	1.383	0.168
	Acting with awareness	−0.058	−0.184, 0.067	−0.917	0.36
	Non reactivity	0.036	−0.074, 0.145	0.637	0.525
Environmental	Observe	0.019	−0.138, 0.176	0.238	0.812
	Describe	0.137	−0.166, 0.44	0.894	0.373
	Non-judging	−0.139	−0.31, −0.032	−1.597	0.112
	Acting with awareness	0.014	−0.148, 0.176	0.176	0.861
	Non reactivity	0.046	−0.184, 0.276	0.395	0.694

## Data Availability

Data are available on request.
